# Involving High School Students in Computational Physics University Research: Theory Calculations of Toluene Adsorbed on Graphene

**DOI:** 10.1371/journal.pone.0159168

**Published:** 2016-08-09

**Authors:** Jonas Ericsson, Teodor Husmark, Christoffer Mathiesen, Benjamin Sepahvand, Øyvind Borck, Linda Gunnarsson, Pär Lydmark, Elsebeth Schröder

**Affiliations:** 1 Hulebäcksgymnasiet, Idrottsvägen 2, SE-435 80 Mölnlycke, Sweden; 2 Randaberg videregående skole, Grødemveien 70, NO-4070 Randaberg, Norway; 3 Microtechnology and Nanoscience, MC2, Chalmers University of Technology, SE-412 96 Göteborg, Sweden; University of Calgary, CANADA

## Abstract

To increase public awareness of theoretical materials physics, a small group of high school students is invited to participate actively in a current research projects at Chalmers University of Technology. The Chalmers research group explores methods for filtrating hazardous and otherwise unwanted molecules from drinking water, for example by adsorption in active carbon filters. In this project, the students use graphene as an idealized model for active carbon, and estimate the energy of adsorption of the methylbenzene toluene on graphene with the help of the atomic-scale calculational method density functional theory. In this process the students develop an insight into applied quantum physics, a topic usually not taught at this educational level, and gain some experience with a couple of state-of-the-art calculational tools in materials research.

## Introduction

In western societies, citizens are often not aware of research activities at universities and other research institutions. This is a problem for a democratic society: research is often to some degree funded by the government, and results have bearings on how we live our lives, either applied directly or in how we perceive the world. Lack of science awareness affects how we, as citizens, can discuss and make collective decisions.

It is important to convey research through education and outreach. One way to increase the public awareness is to let citizens participate in scientific research. School children and youth are natural targets for this as they are already engaged in learning. Their experience from research exposure or participation in younger ages will form their view on research also in later phases of their lives. Science centers and open-house days are ways to reach many people efficiently. Another way is to invite students to directly participate in research.

We here report on computational physics research, in which a group of four senior-year high school students participated. The students participated as part of a course at their school, with a general introduction to research, given by their school, a one week visit to the research group, some months’ work in class, and a final report and poster session for classmates, parents, teachers, and research supervisors.

The high school students participating in this work were all enrolled in a national program at the Swedish “gymnasium”. Enrollment in the program, and choices of courses to take within the program, is by consent of the guardians of the students, when students are underage. This consent is given by signing the application forms electronically. In their final year the students are required to perform a final exam project. At Hulebäcksgymnasiet one option is to participate in a researcher course and there carry out a scientific research project, as described here. The students in the course are informed in advance that they will be carrying out scientific research as part of the course, with all what that encompasses, including dissemination of the results if the scientific project advisers deem this to be realistic, e.g., as the previous students participating in the work leading to Ref [1]. By electing this course as their final exam project the students (and their guardians) leave their consent to such dissemination. In the present project all students reached their age of maturity before or during the course, and accepted in writing the submission for publication of this specific manuscript.

In the specific science project focused on here, the students carry out density functional theory (DFT) calculations to obtain the adsorption energy and structure of the toxic methylbenzene molecule toluene on graphene.

**Fig 1:**
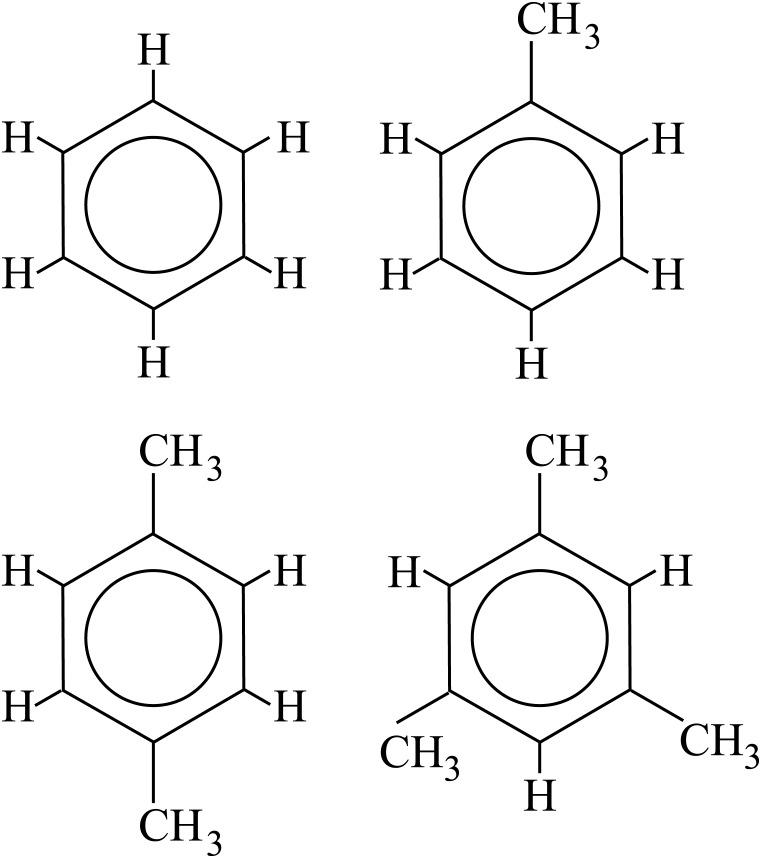
The atomic structures of benzene, and the methylbenzenes toluene, para-xylene, and mesitylene. The student research project focuses on toluene (top right), whereas the continuation of the project involves also benzene and para-xylene (bottom left), the so-called BTX-family, as well as mesitylene (bottom right).

The choice of research topic is of course essential for successfully engaging the students. Understanding toxicity in our environment has proven one such focus that interests our visitors. Methylbenzenes is a group of small, aromatic molecules that are volatile and hazardous. They are benzene molecules with one or more methyl groups attached. For the student collaboration we focus on toluene, a methylbenzene with one methyl group, whereas a broader project of the Chalmers research group (reported elsewhere, Ref [2]) includes also benzene and methylbenzenes with two and three methylgroups: para-xylene (1,4-dimethylbenzene) and mesitylene (1,3,5-trimethylbenzene). The atomic structures are shown in Fig 1.

In the Chalmers research group we study how carbon materials and other filter materials may be used for filtering undesirable molecules from water or air [1, 3–6]. In our studies, smooth, defectless graphene is sometimes used as a first, idealized model of a filter, and methylbenzene adsorption on graphene fits naturally into this program. For example, a similar study of chloroform adsorption on graphene was carried out by a preceding student group from the same high school program one year earlier, later extended into a regular chemical physics publication [1]. The student part of the present computational project is trimmed and chosen such as to minimize the computational time needed, and the results are used as a reference for discussions of the necessary resources in such projects.

In the following we discuss in brief the overall research project of methylbenzenes adsorbed on graphene, and then focus on the toluene student project and the implications for the students and their education. In the S1 File we describe the calculational limitations of the student project.

## The Science Problem

Active carbon and similar materials are often used as filter materials for air and water filters. Although defects and impurities play an important role in how active carbon acts as an adsorbent, already the calculated adsorption energy for adsorption on clean, perfect graphene will be an indication of the strength of the adsorption in the filters.

Recently, the use of nanoporous graphene in reverse osmosis was suggested as a cost-effective way to desalinate sea water [7, 8]. The pressure needed during the reverse osmosis is lower with nanoporous graphene compared to the use of conventional polymeric membranes, keeping down the energy consumption of the process. The water molecules pass through the nanopores, leaving the larger salt ions behind in a growing concentration. In this process pollutant molecules may stick to the graphene membrane [9], by which the water is then also cleaned for pollutants.

In the present project a number of methylbenzenes are used as examples of molecules that need to be filtered from water or air. Toluene, top right in Fig 1, is believed to be neurotoxic [10], and thus definitely not suitable in drinking water.

## Method of computation and results

We here focus on the specific student project of toluene adsorption on graphene. All calculations presented here use the calculational method density functional theory [11] (DFT). It is a method for atomic-scale calculations that builds directly on the Schrödinger equation of quantum physics. The input needed for the DFT calculations is in principle only the atomic numbers of the atoms in the system and their approximate atomic positions, e.g., within the molecules or in the surfaces of the system. In practical use a number of approximations are made, and the choice of approximations influence the size and duration of the calculations as well as the quality of the results obtained.

In the present project, carried out by the high school students, the DFT method vdW-DF [12–15] in the version vdW-DF1 [12] is used, as implemented in the code GPAW [16, 17] with ASE [18, 19]. All calculations are carried out in computational clusters at C3SE, at Chalmers University of Technology, within the Swedish National Infrastructure for Computing (SNIC).

**Fig 2:**
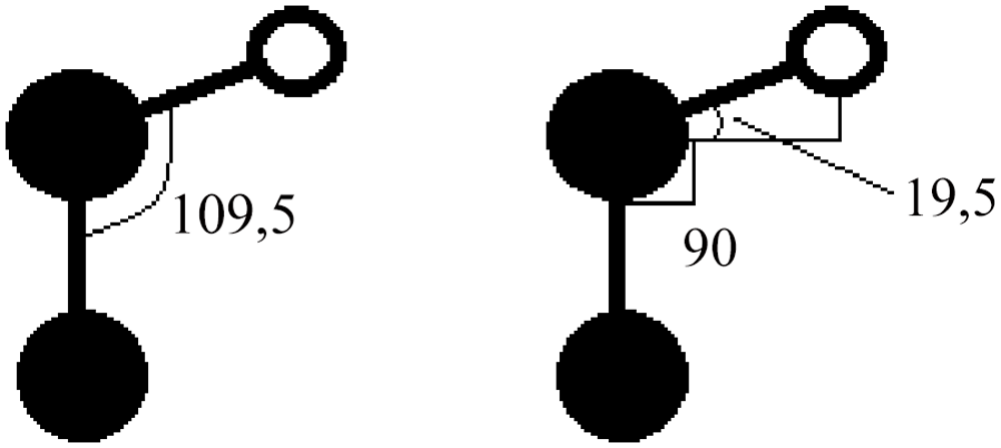
From the student report. Estimating the atomic positions in toluene using symmetry considerations and the bond lengths of C-C and C-H, as well as typical angles (in degrees) of the C-C-H bonds.

Atomic positions for molecules are available from a range of sources online. Since the atomic structure of toluene is relatively simple (Fig 1) the students are instead asked to estimate the atomic positions from knowledge of common C-C and C-H bond lengths and relevant symmetries, Fig 2. The estimated atomic positions are then entered into a fast, but low-quality DFT calculation that moves the atoms such that the remaining net-forces on the atoms are minimized. This changes the atomic positions slightly (and thus the bond lengths and bond angles), and it is therefore not important to start out with precise values for the atomic positions. For this part of the problem the computationally cheap linear-combination-of-atomic-orbitals mode (LCAO) is used. These atomic positions are then used as input for the further calculations, with accurate grid-based wavefunctions [16].

**Fig 3:**
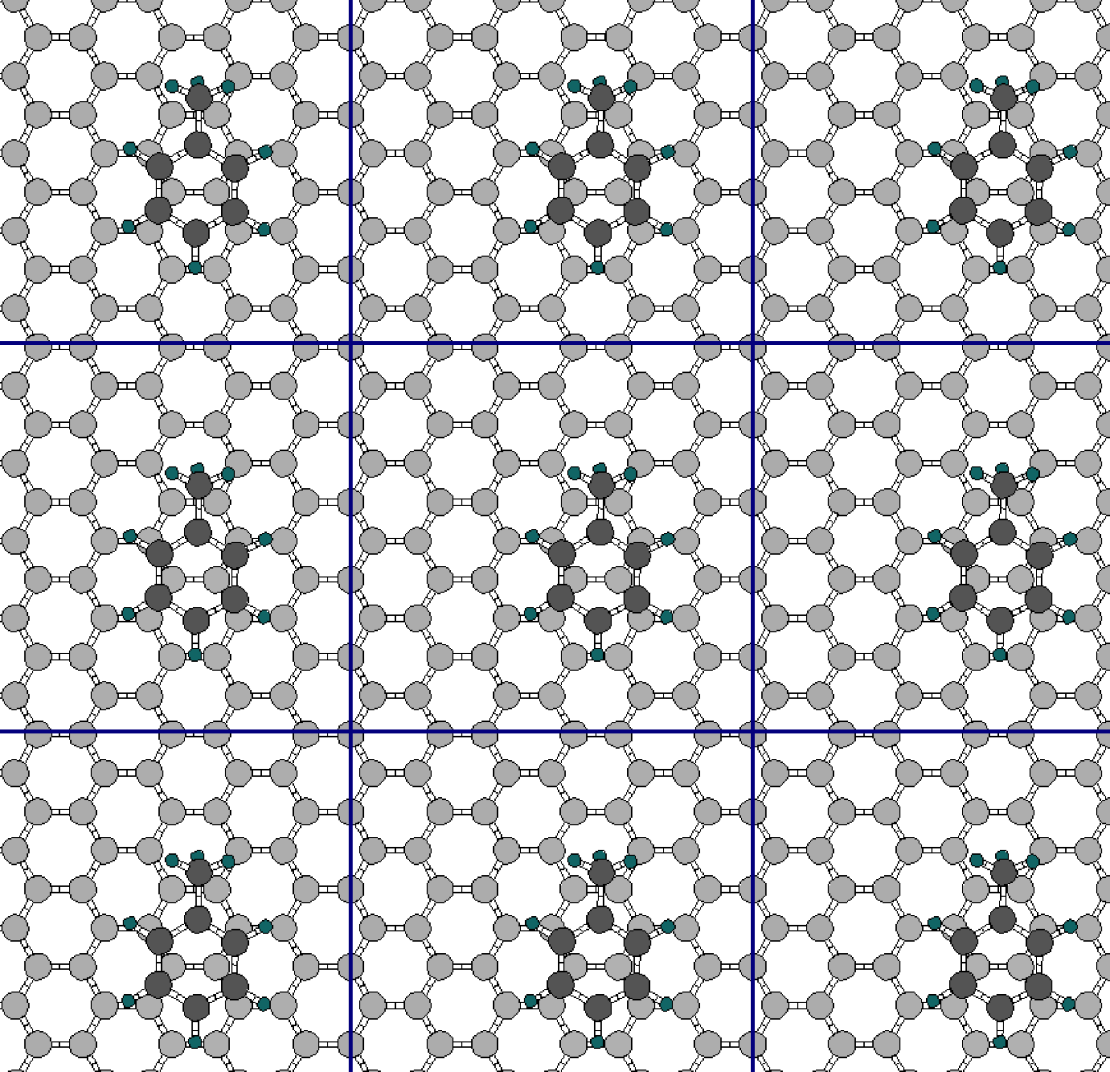
Illustration of the repeated unit cell of the calculations, seen from an angle looking directly down on graphene. The middle rectangle is the limit of one unit cell. The graphene C atoms are light gray large circles, the toluene C atoms are dark gray large circles, and H atoms are small circles. The covalent bonds between atoms are a guide to the eye only, bonds are not explicitly treated in DFT calculations but occur naturally e.g. from the electron density distribution, as in nature. The unit cell is orthorhombic (a box with right angles) with size $3\sqrt{3}a_{g}\times 5a_{g}$ (12.9 Å × 12.4 Å) in the plane of graphene. Here, *a*_*g*_ = 2.48 Å is the lattice parameter of the primitive graphene unit cell in our calculations. The size of the unit cell in the direction perpendicular to the plane of graphene is 23 Å.

The system used for the toluene-on-graphene calculations includes 60 C atoms of graphene and the 15 atoms of toluene. However, in the calculations the unit cell is periodically repeated, so that the graphene is in effect infinite. The toluene molecule is also periodically repeated, and thus there is a sparse network of toluene molecules at fixed separation (equal to the side lengths of the unit cell) on graphene. This is illustrated in Fig 3.

The toluene adsorption energy, *E*_*a*_, is calculated as the difference in total system energy between the situation with the adsorbed molecule and the situation with toluene “far” away from graphene (in reality 11.5 Å away)

$$E_{a}=-\left(E_{\text{bind}}-E_{\text{far}}\right)$$ (1)

where positive values are obtained if toluene binds.

In the S1 File we describe the simplifications used in the student project in order to shorten the student calculations, so that they fill only the available time (one week at the university campus). Together, all simplifications reduce week-long calculations to calculations of 10–30 minutes, albeit at the loss of some of the accuracy. As will be discussed further below, the accuracy is still sufficient for the students to obtain meaningful results.

The adsorption energies *E*_*a*_ are calculated from Eq (1) and are shown in Table 1. We see that for the particular configuration (one methyl H atom pointing down, “methyl corner”) the *E*_*a*_ of the student calculations deviates from the more accurate, but also more costly calculations of the same configuration, by 0.01–0.02 eV (1–2 kJ/mol), or less than 4%. While the accuracy of the student results is not quite publication quality, the effect on the binding energy is marginal, and shows us that such short calculations can indeed be used in student projects and still give realistic results.

**Table 1:** Adsorption energies *E*_*a*_ for toluene on graphene. Calculated with various values of parameters, of real-space grid points (gpts), number of Brillouin zone k-points (kpts), and exponent of energy convergence threshold *n*, 1.5 ⋅ 10^−*n*^ eV/electron. The student research project provided the “student” data for toluene, whereas the medium- and high-quality toluene data were obtained later by the research group [2]. As a comparison, results of experimental measurements from the literature [20, 21] are also shown, however, these results cannot be directly compared, as discussed in the main text.

	gpts	kpts	*n*	*E*_*a*_ [eV]
*methyl corner*				
student	60 × 60 × 96	1 × 1 × 1	6	0.48
medium qual.	108 × 100 × 192	2 × 2 × 1	6	0.50
high quality	108 × 100 × 192	4 × 4 × 1	7	0.49
*methyl edge*				
medium qual.	108 × 100 × 192	2 × 2 × 1	6	0.52
exper., Ref [20]				0.71±0.07
exper., Ref [21]				0.52

**Fig 4:**
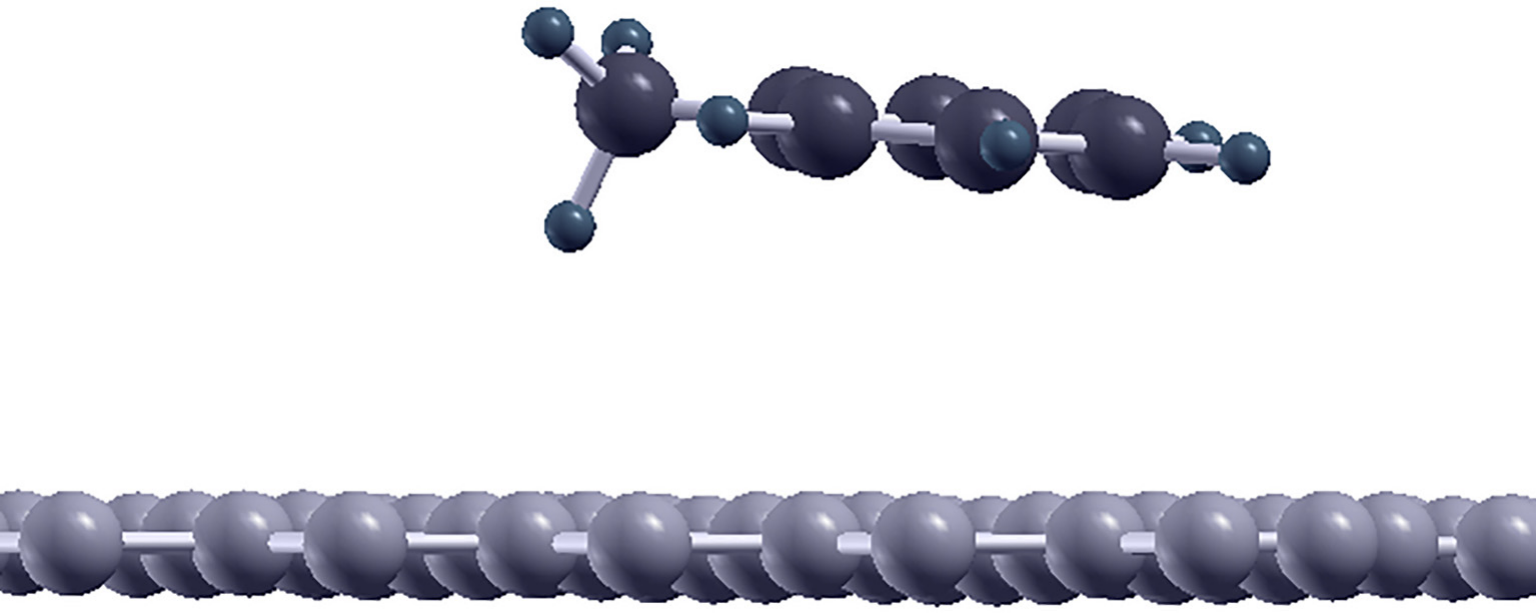
Illustration of the toluene molecule adsorbed on graphene, seen perpendicular to the plane of graphene. Here the configuration found by the students is shown, with one H atom of the methyl group pointing towards graphene, and the other two methyl-group H atoms pointing away (termed “methyl corner” in the text and table).

The students used a configuration where one of the methyl-group H atoms points towards graphene and the other two point away. The triangle formed by the three H atoms in the methyl group thus points one of its corners towards graphene. The configuration is therefore termed “*methyl corner*” in Table 1, and is illustrated in Fig 4. In the subsequent work of the research group [2] configurations with instead two of the methyl-group H atoms pointing towards graphene and the third pointing away (here termed “*methyl edge*”, because an edge of the H atom triangle points to graphene) was found to be slightly more favorable for the adsorption energy. However, even compared to the *methyl edge* configuration the student calculations differ in energy by less than 6% (Table 1).

**Fig 5:**
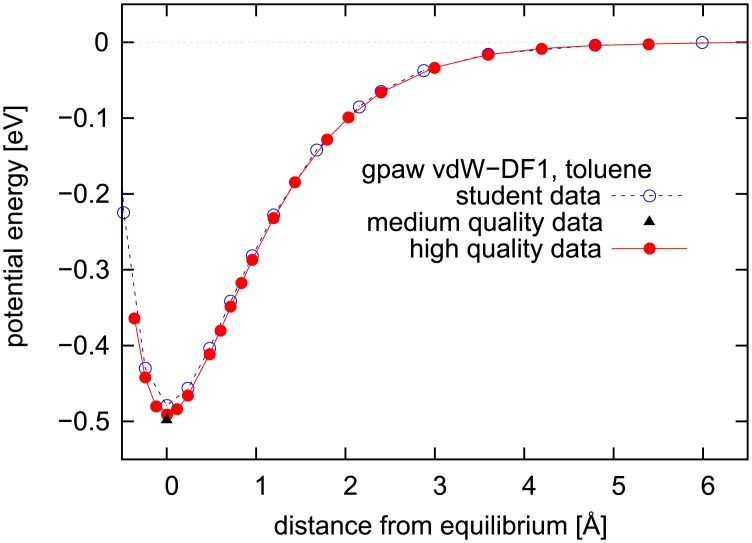
Potential energy curve for toluene flat on top of graphene at various separations. Curves for high quality data as well as the student-produced data with lower quality on the calculational parameters, as discussed in the main text. All data are for the configuration with one methylgroup H atom pointing towards graphene (“methyl corner”).

To refine this discussion, we also include the interaction energy (potential energy curve) of toluene above graphene at various distances, shown in Fig 5. The students carried out the calculations that are marked with open dots, while the highest quality calculations are shown with filled dots. An intermediate-quality calculation, improving only partly on the student settings, is shown as a triangle, and interestingly this intermediate calculation shows results slightly away from both the student results and the high-quality results. This illustrates the fact that the path to convergence in parameter setting is not necessarily monotonous, and for full convergence care must be taken. In the adsorption situation toluene is slightly tilted with respect to the graphene plane (as seen in Fig 4), thus in Fig 5 we use the molecule center of mass for indicating the distance to graphene.

Experimental measurements [20, 21] of the heat of adsorption are also included in Table 1. The experimental results cannot be directly compared to the calculated results for a number of reasons, but they do indicate that the calculated results are reasonable. Contrary to the calculations, the experiments report on results from systems that are not in vacuum, are at non-zero temperature, and are more densely packed at the surface, with a close to one monolayer (ML) coverage. The 1 ML coverage in experiment is to be contrasted with the approximately 0.24–0.29 ML coverage in our calculations, that are further treated such as to obtain the values for a single molecule on graphene (see the S1 File). We estimate the coverage with the help of two sources: Ref [21] reports that the area occupied by an adsorbed toluene molecule on graphite is measured to be 46 Å^2^, of which we have one per 12.9 Å × 12.4 Å area, leading to a coverage of 46/160 ≈ 0.29 ML. Ref [9] finds from molecular dynamics calculations that the coverage concentration is 4.30 *μ*mol/m^2^, which means that the area per molecule is 38.6 Å^2^ and which would mean that our coverage is 0.24 ML. We further know from other similar systems [6] that the adsorption energy increases with coverage, and so the adsorption energy is expected to be larger at a full monolayer coverage.

Our results do not include the zero-point (quantum mechanical) fluctuations, i.e., the quantum mechanical vibrations that are present even at zero temperature. Based on a parabola fit to the potential energy curve in Fig 5 we estimate the zero-point motion perpendicular to graphene to contribute a few meV to the total zero-point motion of toluene on graphene. Assuming that the lateral parts contribute similar amounts, the change in calculated adsorption energies is barely noticeable. We therefore ignore the zero-point fluctuations here.

Finally, another difference to the experiments is that the experiments are adsorption on graphite, which is a stack of sheets of graphene, and not on a single layer, graphene. The effect from the lower-lying layers in graphite is thus not included in our graphene calculations. It has previously been estimated that adsorption on graphite leads to roughly 3% higher interaction energy compared to the same adsorption in graphene [3]. Altogether, the 8–30% difference between experiments and theory is reasonable.

## Project context and preconditions

With an increasing pressure on university faculty to publish more, obtain more external funding, and teach more efficiently, it is no wonder that reaching out to society may seem like an additional burden in a tightly scheduled work day. Nevertheless, outreach is important, and can often be formed such that it ends up benefiting also the faculty member, in addition to the public. Reaching out to school children and other youth, like high school students and undergraduates, has a special value because they already are in a learning process.

Research with undergraduates is well established in the science, technology, engineering, and mathematics (STEM) field. Involving school children and high school youth, on the other hand, is less common, and the barriers are considered significant. In fact, involving high school youth in the STEM field seems to pose similar barriers to those for undergraduate research in the humanities, eloquently described by History professor C. R. Corley in Ref [22]. Including these groups of students in research work is seen as a use of time that is not really available. Exceptions exist, for example large-scale simple experiments like the international Tea Bag Index [23] used with Swedish school children, the best-before-date food study in the fridges of Swedish homes by school children [24], and similar initiatives through the Researchers Night [25]. In these projects a large number (hundreds) of pupils contribute to real research by adding their data points, like the degree of tea leave degradation after months embedded in soil, or the temperatures of the family fridge at various positions and the condition of the food.

In literature on undergraduate research the importance of being able to contribute to original research is emphasized. In Ref [26] the main characteristics of undergraduate research are listed as mentorship, originality, acceptability, and dissemination. For research with younger students, it is natural to expect the conditions to be fulfilled to a lesser degree, but encompassing all four elements makes for a real research experience.

The student project presented here is a project for four high school students, as part of a course on research, given by the high school (Hulebäcksgymnasiet). The course involves a general introduction to science research, measurements in class for a small example project (bouncing ball), a week in a research group on university campus with members of the research group, and a possibility of further contact with the faculty member before and after the week-long stay, if needed. Finally, the students write a report and organize a poster presentation.

The four main characteristics of undergraduate research, mentioned by Osborn and Karukstis [26], are to varying degree also covered by the type of high school research described here: Prior to the week on campus, the research project is introduced (short visit or email) and relevant reading material is suggested. During the first day of the one-week visit, focus is on helping the students navigate the computer codes, understand (to some degree) how they work, and getting a very short introduction to the theory behind the project, in this case quantum physics and density functional theory. As the days go by, the students are expected to work increasingly independent. Thus the *mentoring* has the characteristics of teaching in the beginning of the week, but crosses over to mentoring of more independent nature towards the end of the week. Typically, the students will have the possibility to choose direction of the project after the initial results are in. In the present project the students chose to calculate the full potential-energy curve presented in Fig 5, after having obtained the adsorption energy and successfully having translated between the various units involved, from J/mol to eV, and jumping between length units of Ångström and Bohr, in order to compare results to the literature.

Making sure that the research is *original* is the responsibility of the faculty member, and should not be of consideration for the students until they reach a point where they suggest further calculations beyond the initial results. The research project in itself may be an *acceptable* project from the research community point of view, but care needs to be taken that the quality of the results is also acceptable. This may pose problems with the one-week time frame, but as discussed in the S1 File, in the present project “clever” choices of parameters can lower the time needed without significant loss of accuracy, and results can be verified later with higher accuracy by the faculty participants.

The *dissemination* of the students’ results is mainly via the two student products: the school report and the poster. In some cases the results are also published in scientific journals, after further work by the faculty mentors [1].

The students involved in these projects do this as an alternative way of carrying out their high school diploma project, offered as a special course called Forskarkursen (“The science course”) at the high school Hulebäcksgymnasiet. Yearly 16–24 students are given the opportunity to apply to the course. Students are not selected based on their grades, but it is a prerequisite to be highly motivated and to have the ability to grasp the complexness of science projects.

The course starts at the high school a few months prior to the university project week with one hour of lesson per week. Here the students are informed about the academic system, trained to read scientific papers and discuss ethic scientific dilemmas. In addition, the students perform a small, open investigation answering the question “How many times does a ball bounce?” to illustrate the scientific method when faced with a problem that has no given answer.

After the project week at the university the students analyze their data and write their scientific report with guidance of the high school teachers and the university supervisor. The course has been given for more than ten years, and the course evaluations have always been very positive. The students especially appreciate that they get to perform real research that is of immediate interest for a research group. They are also encouraged by the fact that they actually manage to understand the essence of the specific research field they are working in, even though it is well above their high school science level. Examples of such research projects, besides the one described in this paper, are how shorthorn sculpin react to temperature changes (Department of Zoology, University of Gothenburg), yeast cell responses to oxidative stress (Department of Chemistry and Molecular Biology, University of Gothenburg), gene expression of enterotoxigenic Escherichia coli (Department of Microbiology and Immunology, Sahlgrenska Academy, University of Gothenburg), and visualization of 1P and 2P fluorescence (Department of Physics, University of Gothenburg).

The one-week research-group visit focuses on carrying out the research for the project, but time is also spent seeing the working place and feeling part of the work environment. The roles of the teachers and mentors are relatively clear: the faculty mentor works with the students on campus, whereas the high school teachers work with the students on general areas in school. The high school teachers involved in this course both have a background as researchers in the STEM field, which benefits the interaction between the high school teachers and the university mentors.

The overall content of the project must be carefully chosen. One must be certain to obtain usable results (i.e., negative results should still be of interest, in order to not discourage the students), thus research that measures a number is a better choice than testing a hypothesis that is only interesting if positive. It must be something new, but the group should still be able to easily compare at least some of the results to previous work/experiments in the area.

For the above reason, the present project is similar to previous projects that have been carried out in the research group: it studies the adsorption of small, flat molecules onto a flat surface. Previous such research results, using the same or similar computational tools, include calculations of benzene, adenine, phenol, and the other nucleobases on graphene [3, 4, 27, 28]. From a calculational point of view toluene has the advantage of being a rather small molecule with a relatively stiff part in the aromatic ring. This keeps the need for calculational power and time down, compared to studies of more complicated adsorbants.

On the practical, computational side of the project, the DFT code (GPAW [16, 17]) and the python computational environment (ASE [18, 19]) are open-source codes and as such carry the benefit that the students at any time can download the codes and play around with them at their own computers. A national computational cluster is used for parallelized computations in order to obtain results in a reasonable time, but smaller such calculations can also be carried out at the laptops of the students, given sufficient patience. The fact that this is possible (even if not actually downloaded and installed) helps to demystify the research process.

## Summary

We present an example of organized research for high school students, given as a course at the Swedish high school Hulebäcksgymnasiet, in collaboration with local universities. A specific research project within computational physics is discussed both from the outreach and the scientific point of view.

## Supporting Information

S1 File Simplifications in student calculations. We describe how the student calculations are simplified, to allow for the calculations to finish in the one week available for the students, at the same time keeping a reasonable quality of the results.
(PDF)

## References

[1] Åkesson J, Sundborg O, Wahlström O, Schröder E. A van der Waals density functional study of chloroform and other trihalomethanes on graphene. J Chem Phys. 2012;137(17):174702 10.1063/1.4764356 23145737

[2] Borck Ø, Schröder E. Methylbenzenes on graphene. arXiv:1607.05107

[3] Chakarova-Käck SD, Schröder E, Lundqvist BI, Langreth DC. Application of van der Waals Density Functional to an Extended System: Adsorption of Benzene and Naphthalene on Graphite. Phys Rev Lett. 2006;96(14):146107 10.1103/PhysRevLett.96.146107 16712103

[4] Chakarova-Käck SD, Borck Ø, Schröder E, Lundqvist BI. Adsorption of phenol on graphite (0001) and *α*-Al_2_O_3_ (0001): Nature of van der Waals bonds from first-principles calculations. Phys Rev B. 2006;74(15):155402 10.1103/PhysRevB.74.155402

[5] Londero E, Karlson EK, Landahl M, Ostrovskii D, Rydberg JD, Schröder E. Desorption of n-alkanes from graphene: a van der Waals density functional study. J Phys: Condens Matter. 2012;24(42):424212 10.1088/0953-8984/24/42/424212 23032797

[6] Schröder E. Methanol adsorption on graphene. J Nanomater. 2013;2013:871706 10.1155/2013/871706

[7] Thiel GP. Salty solutions. Physics Today. 2015;68:66 10.1063/PT.3.2828

[8] Surwade SP, Smirnov SN, Vlassiouk IV, Unocic RR, Veith GM, Dai S, et al Water desalination using nanoporous single-layer graphene. Nat Nanotechnol. 2015;10:459 10.1038/nnano.2015.37 25799521

[9] Klomkliang N, Do DD, Nicholson D. Affinity and packing of benzene, toluene, and p-xylene adsorption on a graphitic surface and in pores. Ind Eng Chem Res. 2012;51:5320 10.1021/ie300121p

[10] White RF, Proctor SP. Solvents and neurotoxicity. Lancet. 1997;349:1239 10.1016/S0140-6736(96)07218-2 9130958

[11] Kohn W. Electronic structure of matter—Wave functions and density functionals. Nobel Lecture. Rev Modern Phys. 1999;71:1253.

[12] Dion M, Rydberg H, Schröder E, Langreth DC, Lundqvist BI. van der Waals Density Functional for General Geometries. Phys Rev Lett. 2004;92(24):246401 10.1103/PhysRevLett.92.246401 15245113

[13] Dion M, Rydberg H, Schröder E, Langreth DC, Lundqvist BI. Erratum: van der Waals Density Functional for General Geometries [Phys. Rev. Lett. **92**, 246401 (2004)]. Phys Rev Lett. 2005;95(10):109902 10.1103/PhysRevLett.95.109902 15245113

[14] Thonhauser T, Cooper VR, Li S, Puzder A, Hyldgaard P, Langreth DC. van der Waals density functional: Self-consistent potential and the nature of the van der Waals bond. Phys Rev B. 2007;76(12):125112 10.1103/PhysRevB.76.125112

[15] Berland K, Cooper VR, Lee K, Schröder E, Thonhauser T, Hyldgaard P, et al van der Waals forces in density functional theory: a review of the vdW-DF method. Rep Prog Phys. 2015;78:066501 10.1088/0034-4885/78/6/066501 25978530

[16] Open-source, grid-based PAW-method DFT code GPAW; 2016. http://wiki.fysik.dtu.dk/gpaw/.

[17] Enkovaara J, Rostgaard C, Mortensen JJ, Chen J, Dułak M, Ferrighi L, et al Electronic Structure Calculations with GPAW: A Real-Space Implementation of the Projector Augmented-Wave Method. J Phys: Condens Matter. 2010;22(25):253202 10.1088/0953-8984/22/25/253202 21393795

[18] Python-based atomic simulation environment ASE; 2016. https://wiki.fysik.dtu.dk/ase/.

[19] Bahn SR, Jacobsen KW. An Object-Oriented Scripting Interface to a Legacy Electronic Structure Code. Comput Sci Eng. 2002;4(3):56 10.1109/5992.998641

[20] Ulbricht H, Zacharia R, Cindir N, Hertel T. Thermal desorption of gases and solvents from graphite and carbon nanotube surfaces. Carbon. 2006;44:2931 10.1016/j.carbon.2006.05.040

[21] Monkenbusch M, Stockmeyer R. Neutron Scattering Study on Three Different Phases of Toluene Adsorbed on Graphite Surfaces. Ber Bunsenges Phys Chem. 1981;85(5):442 10.1002/bbpc.19810850520

[22] Corley CR. From Mentoring to Collaborating: Fostering Undergraduate Research in History. History Teacher. 2013;46:397.

[23] Keuskamp JA, Dingemans BJJ, Lehtinen T, Sarneel JM, Hefting MM. Tea Bag Index: A novel approach to collect uniform decomposition data across ecosystems. Methods Ecol Evolut. 2013;4:1070 10.1111/2041-210X.12097

[24] Marklinder I, Eriksson MK. Best-before date—food storage temperatures recorded by Swedish students. Br Food J. 2015;117(6):1764 10.1108/BFJ-07-2014-0236

[25] Forskarfredag; 2016. Available from: http://forskarfredag.se/researchers-night/.

[26] Osborn JM, Karukstis KK. The benefit of Undergraduate Research, Scholarship, and Creative Activity In: Boyd MK, Wesemann J, editors. Broadening Participation in Undergraduate Research: Fostering Excellence and Enhancing the Impact. Washington D.C: Council on Undergraduate Research; 2009 p. 41.

[27] Berland K, Chakarova-Käck SD, Cooper VR, Langreth DC, Schröder E. A van der Waals density functional study of adenine on graphene: Single-molecular adsorption and overlayer binding. J Phys: Condens Matter. 2011;23(13):135001 10.1088/0953-8984/23/13/135001 21403239

[28] Le D, Kara A, Schröder E, Hyldgaard P, Rahman TS. Physisorption of nucleobases on graphene: A comparative van der Waals study. J Phys: Condens Matter. 2012;24(42):424210 10.1088/0953-8984/24/42/424210 23032709

