# Supplementary material for: Involving High School Students in Computational Physics University Research: Theory Calculations of Toluene Adsorbed on Graphene
Source: PLoS One. 2016 Aug 9;11(8):e0159168. doi: 10.1371/journal.pone.0159168 (PMC4978446; doi:10.1371/journal.pone.0159168)
Supplement: S1 File — We describe how the student calculations are simplified, to allow for the calculations to finish in the one week available for the students, at the same time keeping a reasonable quality of the results. (PDF) [file pone.0159168.s001.pdf]

Supporting Information text S1

Supporting information to:
"Involving High School Students in University Research: Calculations of Toluene Adsorbed on Graphene"
PLOS ONE 2016

Authors and affiliations:
Jonas Ericsson[1], Teodor Husmark[1], Christoffer Mathiesen[1], Benjamin Sepahvand[1], Øyvind Borck[2], Linda Gunnarsson[1], Pär Lydmark[1], Elsebeth Schröder[3]
1 Hulebäcksgymnasiet, Idrottsvägen 2, SE-435 80 Mölnlycke, Sweden
2 Randaberg videregående skole, Grødemveien 70, NO-4070 Randaberg, Norway
3 Microtechnology and Nanoscience, MC2, Chalmers University of Technology, SE-412 96 Göteborg, Sweden

Simplifications in the student calculations:

The student calculations presented in the main text were made possible by simplifying the calculations in certain respects. The simplifications made the calculations sufficiently fast for the student project, at the cost of loosing some accuracy otherwise expected from similar research calculations. This supporting information text describes the simplifications and discusses the effect on the results.

The adsorption system is described within an orthorhombic unit that is periodically repeated in all directions. Each toluene molecule is therefore affected by the presence of the neighboring toluene molecules. However, the calculations of binding energy are carried out so as to cancel most of this possible contribution to the energy, yielding the binding energy of a single toluene molecule on graphene.

The density functional theory (DFT) density of the electrons (and the corresponding quantum-mechanical wavefunctions) are described on a uniform grid. The distance between grid points in the student project is almost double that in the research project. Thus the student project only has 17% of the grid points compared to the research projects.

One further difference is the use of only one k-point (the Γ point) in the student calculations whereas for the research calculations we use $(2 \times 2 \times 1)$ or $(4 \times 4 \times 1)$ k-points. The k-points are the discretization of the reciprocal space, used for easy evaluation of a double gradient in the kinetic energy. The use of only the Γ point is common, and correct, in calculations of material systems that are not extended, e.g., in the interaction of two small molecules. Here one part of the system is confined (toluene) and the other part is extended (graphene). Thus it is not unreasonable to use the Γ point only, although for accuracy research calculations usually do make use of a more dense grid of k-points. By use of time reversal symmetry $(4 \times 4 \times 1)$ k-points can be reduced to 4 points in the irreducible Brillouin zone, four times more than just the Γ point.

Since the computational need grows faster than linearly with number of grid points and number of irreducible k-points the student calculations can be carried out with (much) less than 4% (1/4 of 17%) of the resources required for the high-quality calculations. This reduces otherwise week-long calculations on the computational cluster to few-hour calculations on less computations nodes, making the project attainable for a one-week student project.

Finally, the high-quality calculations differ from the student (and medium-quality calculation) by a stricter convergence condition on the energy calculated: the calculation is considered converged if changes are less than $1.5 \cdot 10^{-n}$ eV/electron, where the requirement is $n = 7$ for the high-quality but $n = 6$ for the medium-quality and student calculations. This does change the computational time needed, but much less than the other sources above, and this difference will be ignored here.
